# Medicinal and Edible Fungi as an Alternative Medicine for Treating Age-Related Disease

**DOI:** 10.1155/2014/638561

**Published:** 2014-04-29

**Authors:** Da-wei Qin, Chunchao Han

**Affiliations:** ^1^School of Chemistry and Pharmaceutical Engineering, Qilu University of Technology, Jinan 250353, China; ^2^School of Pharmacy, Shandong University of Traditional Chinese Medicine, Jinan 250355, China

Age-related diseases such as type 2 diabetes, cardiovascular disease, and cancer involve epigenetic modifications, where accumulation of minute changes in the epigenome over time leads to disease manifestation. Epigenetic changes are influenced by life style and diets. This represents an avenue whereby dietary components could accelerate or prevent age-related diseases through their effects on epigenetic modifications. Therefore, new therapeutic approaches are needed to treat them more efficiently.

The lack of effective and widely applicable pharmacological treatments for ischemic stroke patients may explain a growing interest in traditional medicines. Some of basidiomycetous fungi produce substances with potential medical effects and are called medicinal mushrooms. Fungi (yeasts, molds, and mushrooms) have diverse morphological, physiological, and ecological characteristics that support their diverse lifestyles. These specific interspecies interactions depend on the production of a wide range of bioactive substances. Higher fungi are well recognized for their medicinal properties and have been used in traditional medicine for millennia. The medicinal effects attributed to fungi, based mainly on uncharacterized substances or extracts, include antiviral, immunomodulatory, antitumor, antioxidant, radical scavenging, anti-inflammatory, antihyperlipidemic or antihypercholesterolemic, hepatoprotective, and antidiabetic effects.

*Cordyceps sinensis* (CS) has been used as a tonic for longevity, endurance, and vitality for thousands of years by the Chinese. Many studies have shown that CS regulates insulin sensitivity and decreases plasma cholesterol levels. W. Qi et al. investigated the protective effect of cordymin on diabetic osteopenia in alloxan-induced diabetic rats and the possible mechanisms involved. The diabetic rats received daily intraperitoneal injection with cordymin (20, 50, and 100 mg/kg/day) for 5 weeks. Cordymin could restore the circulating blood glucose, glycosylated hemoglobin (HbA1c), serum alkaline phosphatase (ALP), tartrate resistant acid phosphatase (TRAP), and insulin levels in a dose-dependent manner. Also, the treatment of diabetic rats with cordymin could partially reverse the *β* cells death and decrease the total antioxidant status (TAOS) in the diabetic rats. The results may directly and indirectly account for the possible mechanism of the beneficial effect of cordymin on diabetic osteopenia.

The basidiomycete* Agaricus blazei* Murill (AbM), popularly known as “sun mushroom,” is native to Brazil and widely grown in Japan and China because of its medicinal properties. It is widely used for nonprescript, medicinal purposes, both as an edible mushroom and in the form of extracts. AbM has traditionally been used for the prevention of a range of diseases, including cancer, hepatitis, atherosclerosis, hypercholesterolemia, diabetes, and dermatitis. P. Wang et al. investigated the anti-inflammatory activity of water-soluble polysaccharide of* Agaricus blazei *Murill (WSP-AbM) on ovariectomized osteopenic rats. The rats were administered orally WSP-AbM (200 mg/kg BW) for 8 weeks. Subsequent serum maleic dialdehyde (MDA) level, total antioxidant status (TAOS), nuclear factor kappa B (NF-*κ*B) level, polymorphonuclear (PMN) cells level, interleukin-1*β* (IL-1*β*) level, inducible nitric oxide synthase (iNOS) level, tumor necrosis factor-*α* (TNF-*α*) level, adhesion molecule (ICAM-1), and cyclooxygenase-2 (COX-2) were determined by enzyme linked immunosorbent assay (ELISA) and immunohistochemistry, respectively. WSP-AbM administration markedly decreased serum IL-1*β* and TNF-*α* levels and the expressions of ICAM-1, COX-2, and iNOS NF-*κ*B compared with OVX rats. WSP-AbM administration also markedly decreased PMN infiltration. In conclusion, we observed that WSP-AbM supplementation had anti-inflammatory effects in a model of osteoporosis disease. H. Wang et al. also review* Agaricus blazei* Murill. They demonstrated ABM is useful against a variety of diseases like cancer, tumor, chronic hepatitis, diabetes, atherosclerosis, hypercholesterolemia, and so on. W. Ji et al. examined the neuroprotective effect of* Agaricus brasiliensis* (AbS) against STZ-induced diabetic neuropathic pain in laboratory rats. After 6 weeks of treatments, all animals were sacrificed to study various biochemical parameters. Treatment with AbS 80 mg/kg in diabetic animals showed significant increase in body weight, pain threshold, and paw withdrawal threshold and significant decrease in serum glucose, LPO and NO level, Na-K-ATPase level, and TNF-*α* and IL-1*β* level as compared to vehicle treated diabetic animals in dose and time-dependent manner. AbS can offer pain relief in PDN.

*Ganoderma lucidum* is a popular medicinal mushroom that has been used as a home remedy in traditional Chinese medicine for the prevention or treatment of a variety of diseases including cancer. Today,* G. lucidum* is recognized as a dietary supplement recommended in many countries as a cancer therapeutic. Another important property of this edible mushroom is the ability to take up and accumulate trace metals such as cadmium, lead, arsenic, copper, nickel, silver, chromium, and mercury in the body or mycelium of the mushroom. G. Min-chang et al. compared the effect and toxicity of organic selenium (selenium-enriched protein from* Ganoderma lucidum*) with inorganic selenium (IOSe) in preventing asthma in ovalbumin-induced asthmatic mice. The results showed that the serum Se levels in the mice fed the Pro-Se were significant elevations. It results in restoration of the level of endogenous antioxidant enzyme, lower levels of TNF-*α* and IL-1*β*, and activated NF-*κ*B in the asthmatic mice. Our experiments have demonstrated profound differences between the activities of organic selenium and inorganic selenium in experimental conditions. These data provide an important proof of the concept that organic selenium might be a new potential therapy for the management of childhood asthma in humans.

By compiling these papers, we hope to enrich our readers and researchers with respect to medicinal and edible fungi, yet usually highly treatable fungi for age-related disease.

*Da-wei Qin* *Da-wei Qin* *Chunchao Han* *Chunchao Han*

